# High Prevalence of ST131 Subclades C2-*H*30Rx and C1-M27 Among Extended-Spectrum β-Lactamase-Producing *Escherichia coli* Causing Human Extraintestinal Infections in Patients From Two Hospitals of Spain and France During 2015

**DOI:** 10.3389/fcimb.2020.00125

**Published:** 2020-03-24

**Authors:** Saskia-Camille Flament-Simon, Vanesa García, Marion Duprilot, Noémie Mayer, María Pilar Alonso, Isidro García-Meniño, Jesús E. Blanco, Miguel Blanco, Marie-Hélène Nicolas-Chanoine, Jorge Blanco

**Affiliations:** ^1^Laboratorio de Referencia de E. coli (LREC), Departamento de Microbioloxía e Parasitoloxía, Facultade de Veterinaria, Universidade de Santiago de Compostela (USC), Lugo, Spain; ^2^Grupo Escherichia coli, Instituto de Investigación Sanitaria de Santiago de Compostela (IDIS), Santiago, Spain; ^3^Service de Microbiologie, Hôpital Beaujon, AP-HP, Clichy, France; ^4^INSERM and University Paris Diderot, IAME, UMR 1137, Paris, France; ^5^Unidade de Microbioloxía, Hospital Universitario Lucus Augusti (HULA), Lugo, Spain

**Keywords:** β-lactamases, CTX-M, *E. coli*, ESBL, ExPEC, resistance, ST131, ST1193

## Abstract

The present study was carried out to evaluate the prevalence of sequence type 131 (ST131) among 188 extended-spectrum β-lactamase-producing *Escherichia coli* (ESBL-EC) collected in 2015 in Lucus Augusti University hospital (Lugo, Spain) and AP-HP Beaujon hospital (Clichy, France) with regard to other STs and to characterize, the types of ESBL produced, serotypes, virulence factor (VF)-encoding genes and the ST131 clades and subclades. ST131 was detected in 33 (39.1%) and 46 (47.9%) of the isolates in Lucus Augusti and Beaujon, respectively. The 109 remaining isolates displayed 57 other STs, the following STs being displayed by at least three isolates: ST10 (8 isolates), ST23 (3), ST38 (4), ST58 (3), ST88 (5), ST95 (4), ST167 (3), ST354 (5), ST361 (3), ST410 (6), ST648 (4), ST744 (3), and ST1615 (6). ST354, ST410, and ST1615 were significantly (*P* < 0.05) more frequent in Lucus Augusti (5.4%, 6.5%, and 6.5%) than in Beaujon (0% for the three STs). The new globally emerging clone ST1193 among extraintestinal clinical ESBL-EC was identified in one isolate from France and one from Spain. CTX-M-15 was the commonest ESBL detected in the two hospitals (44.6% in Lucus Augusti and 50.0% in Beaujon). CTX-M-14 was significantly (*P* = 0.0003) more frequent in Lucus Augusti (31.5%) than in Beaujon (10.4%), whereas CTX-M-1 (20.8 vs. 7.6%; *P* = 0.008) and CTX-M-27 (15.6 vs. 6.5%; *P* = 0.0389) were more frequent in Beaujon than in Lucus Augusti. The ST131 isolates showed a higher virulence score (mean 13.367) compared with the non-ST131 isolates (mean 7.661) (*P* < 0.001). Among the 79 ST131 isolates, most of them (52; 65.8%) belonged to subclade C2 (also known as subclone

*H*30Rx) followed by those belonging to subclade C1 (cluster C1-M27: 16 isolates, 20.3%; cluster non-C1-M27: 6 isolates, 7.6%) and clade A (4 isolates; 5.1%). The C2 subclade isolates showed a higher VF-encoding gene score (mean 14.250) compared with the C1-M27 cluster isolates (mean 10.875) (*P* < 0.001). In conclusion, this study highlights the epidemiological differences between the ESBL-EC isolated from two hospitals of France and Spain obtain in 2015 and reports, for the first time, the presence of clone ST1193 in Spain.

## Introduction

Extended-spectrum β-lactamase-producing *Escherichia coli* (ESBL-EC) is an important cause of urinary tract and bloodstream infections, as well as other types of human extraintestinal infections (Arnaud et al., 2015; Mamani et al., 2019). The main sequence type (ST) responsible for the global increase in ESBL-EC is, without a doubt, ST131 (Nicolas-Chanoine et al., 2008, 2014). This pandemic high-risk clone has numerous virulence factor (VF)-encoding genes (Blanco et al., 2013). Whole-genome sequencing (WGS) analysis had revealed that ST131 consists of three different clades (A, B, and C) characterized by different alleles of the *fimH* gene that is implicated in the colonization abilities, i.e., *fimH*41, *fimH*22, and *fimH*30, respectively (Petty et al., 2014; Ben Zakour et al., 2016). Subclade C2 (also known as subclone *H*30Rx) associated with the production of CTX-M-15 seems to be the most expanded and successful ST131 sublineage (Banerjee et al., 2013; Price et al., 2013; Dahbi et al., 2014; Peirano et al., 2014; Sauget et al., 2016). However, cluster C1-M27 that belongs to subclade C1 and produces CTX-M-27 has recently expanded, first in Japan (Matsumura et al., 2016, 2017), then in other countries (Thailand, Australia, Canada, USA, France, Italy, Germany, The Netherlands and Spain) (Blanc et al., 2014; Birgy et al., 2016; Bevan et al., 2017; Merino et al., 2018; Peirano and Pitout, 2019). The other STs frequently found among ESBL-EC are ST10, ST38, ST405, ST410, ST648, and ST1193 (Brisse et al., 2012; Naseer et al., 2012; Peirano et al., 2012; Izdebski et al., 2013; Peirano and Pitout, 2019). Besides there are notable differences with regard to the ESBL-EC epidemiology according to the countries, including, sometimes, the emergence of new lineages. Thus, a new clade of ST131 associated with the production of the CTX-M-101 enzyme and the *fimH*27 allele has recently been identified in *E. coli* responsible for bacteremia in Denmark (Roer et al., 2017).

The present study was carried out to evaluate the prevalence of clone ST131 among ESBL-EC collected in 2015 in Lucus Augusti University hospital (Lugo, Spain) and AP-HP Beaujon hospital (Clichy, France) with regard to the other STs and to characterize the types of ESBL produced, the serotypes, and the VF-encoding genes as well as the clades and the subclades of the ST131 isolates. This study allowed us to highlight the ESBL-EC epidemiological differences in the two hospitals and to report, for the first time, the presence of the new emerging global clone ST1193 among the ESBL-EC clinical isolates from Spain.

This study was presented in part at the 29th European Congress of Clinical Microbiology and Infectious Diseases, Amsterdam, 13–16 April 2019 (Flament-Simon et al., 2019).

## Materials and Methods

In this study, 188 non-duplicate (one isolate per patient) ESBL-EC isolated in 2015 in Spain (92 from Lucus Augusti hospital in Lugo) and in France (96 from Beaujon hospital in Clichy) were characterized. They comprised 139 isolates from urine, 25 from blood, seven from bile, and 17 from various other sources.

The following characters were determined as previously described: serotypes using all available O (O1 to O181) and H (H1 to H56) antisera (Guinée et al., 1981), ESBL types (TEM, SHV, and CTX-M enzymes) (Leflon-Guibout et al., 2004), phylogenetic groups (A, B1, B2, C, D, E, F) (Clermont et al., 2013), STs of Achtman scheme (Wirth et al., 2006), clonotypes (*fumC* and *fimH* genes) (Weissman et al., 2012), and VF-encoding genes (Mamani et al., 2019). Isolates were classified as extraintestinal pathogenic *E. coli* (ExPEC) (Johnson et al., 2015) if positive for ≥2 of 5 markers, including *papAH* and/or *papC, sfa/focDE, afa/draBC, kpsM II*, and *iutA*, as uropathogenic *E. coli* (UPEC) (Spurbeck et al., 2012) if positive for ≥3 of 4 markers, including *chuA, fyuA, vat*, and *yfcV*, and as avian pathogenic *E. coli* (APEC) (Johnson et al., 2008) if positive for ≥4 of 5 markers (*hlyF, iutA, iroN, iss*, and *ompT*). The virotypes A to F were assigned according to the scheme developed by Dahbi et al. (2014). The ST131 clades (A, B, C), subclade C2 (also known as subclone *H*30Rx) and the two clusters of subclade C1 (C1-M27 and the non-C1-M27) were stablished using the PCR assay recently developed by Matsumura et al. (2017).

All the *P*-values were calculated using the Fisher's exact test, except for the comparison of the means that was performed using the one-way ANOVA test. *P* < 0.05 were considered statistically significant.

## Results

### Types of ESBL Enzymes

A total of 89 (47.3%) isolates were positive for CTX-M-15, 39 (20.7%) for CTX-M-14, 27 (14.4%) for CTX-M-1, 21 (11.2%) for CTX-M-27, 11 (5.9%) for SHV-12, two (1.1%) for CTX-M-32 and one (0.5%) for CTX-M-55. CTX-M-15 was the commonest ESBL detected in the two hospitals (44.6% in Lucus Augusti and 50.0% in Beaujon). CTX-M-14 was significantly (*P* = 0.0003) more frequent in Lucus Augusti (31.5%) than in Beaujon (10.4%), whereas CTX-M-1 (20.8 vs. 7.6%; *P* = 0.008) and CTX-M-27 (15.6 vs. 6.5%; *P* = 0.0389) were more frequent in Beaujon than in Lucus Augusti (Table 1).

**Table 1:** Characteristics of 188 ESBL-EC isolated from extraintestinal infections.

**Characteristic**	**No. (%) of isolates**		***P*-value**
	**Lucus augusti hospital, Spain (*n* = 92)**	**Beaujon hospital, France (*n* = 96)**	
**ESBL enzyme**			
CTX-M-1	7 (7.6)	**20 (20.8)**	0.008
CTX-M-14	**29 (31.5)**	10 (10.4)	0.0003
CTX-M-15	41 (44.6)	48 (50.0)	
CTX-M-27	6 (6.5)	**15 (15.6)**	0.0389
CTX-M-32	2 (2.2)	0	
CTX-M-55	0	1 (1.0)	
SHV-12	8 (8.7)	3 (3.1)	
**Phylogenetic group**			
A	**18 (19.6)**	9 (9.4)	0.0367
B1	7 (7.6)	11 (11.5)	
B2	38 (41.3)	**57 (59.4)**	0.0097
C	**16 (17.4)**	6 (6.3)	0.0151
D	1 (1.1)	2 (2.1)	
E	2 (2.2)	8 (8.3)	
F	**10 (10.9)**	3 (3.1)	0.0341
**ST131**			
Total	33 (39.1)	46 (47.9)	
Clade A	1 (1.1)	3 (3.1)	
Subclade C1 Cluster C1-M27	5 (5.4)	11 (11.5)	
Subclade C1 Cluster non-C1-M27	0	**6 (6.3)**	0.0164
Subclade C2	27 (29.3)	25 (26.0)	
Clade-Not typeable	0	1 (1.0)	
Clonotype CH40-30	30 (32.6)	43 (44.8)	
Clonotype CH40-35	2 (2.2)	0	
Clonotype CH40-41	1 (1.1)	3 (3.1)	
Virotype A	**7 (7.6)**	1 (1.0)	0.0280
Virotype C1	1 (1.1)	1 (1.0)	
Virotype C2	10 (10.9)	15 (15.6)	
Virotype C3	0	2 (2.1)	
Virotype E	10 (10.9)	8 (8.3)	
Virotype F	3 (3.3)	**11 (11.5)**	0.0293
Virotype A-like	1 (1.1)	6 (6.3)	
Virotype E-like	1 (1.1)	0	
Virotype-not typeable	1 (1.1)	2 (2.1)	
**Other ST^a^**			
ST10	6 (6.5)	2 (2.1)	
ST23	1 (1.1)	2 (2.1)	
ST38	0	4 (4.2)	
ST58	2 (2.2)	1 (1.0)	
ST88	1 (1.1)	4 (4.2)	
ST95	0	4 (4.2)	
ST167	2 (2.2)	1 (1.0)	
ST354	**5 (5.4)**	0	0.0265
ST361	2 (2.2)	1 (1.0)	
ST410	**6 (6.5)**	0	0.0126
ST648	3 (3.3)	1 (1.0)	
ST744	1 (1.1)	2 (2.1)	
ST1615	**6 (6.5)**	0	0.0126

a *Represented by at least 3 isolated*.

*Significant differences are indicated in bold*.

### Phylogenetic Groups

The most frequent phylogenetic group was B2 (50.5%), followed by A (14.4%), C (11.7%), B1 (9.6%), F (6.9%), E (5.3%), and D (1.6%). Phylogenetic groups A, C and F were found more frequently among Lucus Augusti isolates, while phylogenetic group B2 was more frequent among Beaujon isolates (*P* < 0.05) (Table 1).

Among CTX-M-15 and CTX-M-27-producing isolates, the most frequent phylogenetic group was B2, while among those producing CTX-M-1, CTX-M-14, and SHV-12, the most frequent phylogenetic groups were B1, C, and F, respectively (Table 2).

**Table 2:** Phylogenetic groups and sequence types according to ESBL enzymes.

**ESBL enzyme (no. of isolates)**	**Phylogenetic groups**	**Sequence types**
CTX-M-1 (27)	A (3), B1 (11), B2 (3), C (6), D (1), E (2), F (1)	ST10 (1), ST23 (2), ST34 (1), ST58 (1), ST69 (1), **ST88** (3), ST90 (1), ST131 (2), ST155 (1), ST162 (1), ST205 (1), ST224 (1), ST453 (2), ST1266 (1), ST1431 (1), ST2067 (1), ST2558 (1), ST2766 (1), ST3778 (1), ST8152 (1), ST new 2-6057like (1), ST new 3-1268like (1)
CTX-M-14 (39)	A (8), B1 (4), B2 (10), C (11), E (2), F (4)	**ST10** (4), ST12 (1), ST59 (1), ST73 (1), ST88 (2), ST93 (1), ST95 (2), ST131 (1), **ST167** (3), **ST354** (3), ST357 (1), ST362 (1), ST404 (2), ST405 (1), **ST410** (3), ST448 (1), ST602 (1), ST1154 (1), ST1193 (1), **ST1615** (6), ST5528 (1), ST10328 (1)
CTX-M-15 (89)	A (11), B2 (64), C (4), D (2), E (4), F (4)	ST4 (1), ST34 (1), ST38 (2), ST44 (1), ST69 (1), ST90 (1), ST95 (2), ST127 (1), **ST131** (59), ST141 (1), ST358 (1), **ST361** (3), ST405 (1), **ST410** (3), ST540 (1), **ST648** (4), ST744 (2), ST1284 (1), ST2279 (1), ST3075 (1), ST5214 (1)
CTX-M-27 (21)	B2 (18), C (1), E (2),	**ST131** (17), ST38 (2), ST90 (1), ST1193 (1)
CTX-M-32 (2)	B1 (2)	ST58 (2)
CTX-M-55 (1)	A (1)	ST744 (1)
SHV-12 (11)	A (4), B1 (1), C (1), F (5)	ST10 (2), ST23 (1), ST117 (1), ST156 (1), ST354 (2), ST1485 (1), ST3778 (1), ST new 1-10 like (2)

*The most frequent STs are indicated in bold*.

### Sequence Types

ST131 was detected in 33 (39.1%) and 46 (47.9%) of isolates in Lucus Augusti and Beaujon, respectively. The 109 remaining isolates displayed 57 different STs and the following STs displayed at least three isolates: ST10 (8 isolates), ST23 (3), ST38 (4), ST58 (3), ST88 (5), ST95 (4), ST167 (3), ST354 (5), ST361 (3), ST410 (6), ST648 (4), ST744 (3), and ST1615 (6). ST354, ST410, and ST1615 were significantly (*P* < 0.05) more frequent in Lucus Augusti (5.4, 6.5, and 6.5%) than in Beaujon (0% for the three STs) (Tables 1, 2). The new emerging global clone ST1193 was identified in one isolate from France and one from Spain. The majority (121 of 188; 64.4%) of ESBL-EC isolates belonged to only three clonal complexes: CC10 (19 isolates), CC23 (22 isolates) and CC131 (80 isolates) (Table S1).

### Clonotypes, Clades and Subclades of ST131 Isolates

The 79 ST131 isolates were distributed in three clonotypes: CH40-30 (73 isolates), CH40-35 (2), CH40-41 (4) (Table 1). Subclade C2 (also known as subclone *H*30Rx) was the commonest subclade detected among the 79 ST131 isolates (52 isolates; 65.8%), followed by cluster C1-M27 (16 isolates; 20.3%), cluster non-C1-M27 (6 isolates; 7.6%), and clade A (4 isolates; 5.1%). The 52 C2 subclade isolates were positive for CTX-M-15, whereas the 16 C1-M27 isolates were positive for CTX-M-27. Five non-C1-M27 isolates of C1 subclade were positive for CTX-M-15 and one for CTX-M-14. The four isolates belonging to clade A were positive for CTX-M-1 (2), CTX-M-15 (1), and CTX-M-27 (1).

### Clones

A total of 71 clones (defined by the association of phylogroup, clonotype and ST) were identified among the 188 ESBL-EC with 23 of them including at least two isolates and only five at least five isolates: A-CH11-54-ST10 (6 isolates), B2-CH40-30-ST131 (73), C-CH4-24-ST410 (6), C-CH263-32-ST1615 (6), and F-CH88-58-ST354 (5) (Table 3).

**Table 3:** ESBL enzymes, serotypes and ExPEC, UPEC and APEC status according to the clones including at least two isolates.

**Clones (no. of isolates from Spain and France)**	**ESBL enzymes**	**Serotypes**	**ExPEC**	**UPEC**	**APEC**
A-CH11-54-ST10 (5/1)	CTX-M-14 (4), SHV-12 (2)	O6:HNM (1), O101:HNT (2), ONT:HNM (3)	0	0	2
A-CH11-negative-ST167 (2/1)	CTX-M-14 (3)	O101:HNM (2), O101:H25 (1)	1	0	0
A-CH11-54-ST744 (1/2)	CTX-M-15 (2), CTX-M-55 (1)	ONT:H9 (1), ONT:HNM (2)	0	0	0
A-CH99-54-ST361 (2/1)	CTX-M-15 (3)	O9:HNM (3)	0	0	0
B1-CH4-32-ST58 (1/1)	CTX-M-1 (1), CTX-M-32 (1)	O9:H25 (1), ONT:HNM (1)	1	0	2
B1-CH6-31-ST453 (0/2)	CTX-M-1 (2)	O23:HNM (1), ONT:HNM (1)	0	0	1
B2-CH38-15-ST95 (0/2)	CTX-M-14 (2)	O18:H7 (2)	2	2	2
B2-CH38-294-ST95 (0/2)	CTX-M-15 (2)	O18:H7 (2)	2	2	2
B2-CH40-30-ST131 (30/43)	CTX-M-15 (56), CTX-M-27 (16), CTX-M-14 (1)	O25:H4 (69), O14:H4 (1), ONT:H4 (3)	68	73	0
B2-CH40-35-ST131 (2/0)	CTX-M-15 (2)	O25:H4 (2)	2	2	0
B2-CH40-41-ST131 (1/3)	CTX-M-1 (2), CTX-M-15 (1), CTX-M-27 (1)	O16:H5 (3), O153:H5 (1)	3	2	0
B2-CH14-27-ST404 (1/1)	CTX-M-14 (2)	O75:HNM (2)	2	2	0
B2-CH14-64-ST1193 (1/1)	CTX-M-14 (1), CTX-M-27 (1)	O75:HNM (2)	2	2	0
C-CH4-35-ST23 (1/2)	CTX-M-1 (2), SHV-12 (1)	O55:H9 (1), O78:HNM (2)	1	0	2
C-CH4-41-ST88 (0/2)	CTX-M-1 (1), CTX-M-14 (1)	O8:HNM (1), O86:HNT (1)	2	0	0
C-CH4-303-ST88 (0/2)	CTX-M-1 (2)	O9:H17 (2)	2	0	2
C-CH4-142-ST90 (2/0)	CTX-M-1 (1), CTX-M-15 and CTX-M-27 (1)	O8:H9 (2)	0	0	0
C-CH4-24-ST410 (6/0)	CTX-M-14 (3), CTX-M-15 (3)	O9:HNM (2), O20:H9 (3), ONT:HNM (1)	0	0	0
C-CH263-32-ST1615 (6/0)	CTX-M-14 (6)	O11:H9 (3), O153:H9 (1), ONT:H9 (2)	0	0	0
E-CH26-negative-ST38 (0/3)	CTX-M-15 (1), CTX-M-27 (2)	O86:H18 (3)	3	0	0
E-CH37-27-ST405 (1/1)	CTX-M-14 (1), CTX-M-15 (1)	O102:H4 (1), O102:HNM (1)	0	0	0
F-CH88-58-ST354 (5/0)	CTX-M-14 (3), SHV-12 (2)	O1:H34 (1), O1:HNM (1), O11:H4 (2), O153:HNT (1)	1	2	0
F-CH4-171-ST648 (2/0)	CTX-M-15 (2)	O45:H45 (2)	0	0	0

### Serotypes

The 188 ESBL-EC isolates belonged to 30 O serogroups and expressed 17 different H antigens, but 71 of the 79 ST131 isolates belonged to serotype O25:H4. The other prevalent serotypes were: O9:HNM (three ST361 isolates), O11:H9 (three ST1615 isolates), O16:H5 (three ST131 isolates), O18:H7 (four ST95 isolates), O20:H9 (three ST410 isolates), O75:HNM (two ST404 and two ST1193 isolates of clonal complex 14), and O86:H18 (three ST38 isolates). The H4 and H5 flagellar antigens were associated with ST131, the H7 with ST95, the H9 with ST10, ST744, and four STs of the clonal complex 23 (ST23, ST90, ST410, ST1615), the H18 with ST38 and ST69, and the H6 and the H45 antigens with ST648 (Table 3 and Table S1).

### Virulence Factor (VF)-Encoding Genes

Of the 188 ESBL-EC isolates, 57.4% were classified as ExPEC, 52.7% as UPEC and 12.8% as APEC. The prevalence of ExPEC (92.4 vs. 32.1%) (*P* < 0.001) and UPEC (97.5 vs. 20.2%) (*P* < 0.001) status were higher within ST131 isolates than within non-ST131 isolates. In contrast, the prevalence of APEC (0 vs. 22%) status was higher among non-ST131 isolates (*P* < 0.001) (Table 4).

**Table 4:** Virulence factor-encoding genes in the studied 188 isolates and according to ST131 lineage.

**Genes**	**No. (%) of isolates**					***P-*value^a^**	***P*-value^a^**
	**Total (*n* = 188)**	**ST131 Cluster C1-M27 (*n* = 16)**	**ST131 Subclade C2 (*n* = 52)**	**ST131 (*n* = 79)**	**Non-ST131 (*n* = 109)**	**C1-M27 vs. C2**	**ST131 vs. non-ST131**
**Adhesin**							
*fimH*	180 (95.7)	16 (100)	52 (100)	79 (100)	101 (92.7)		
*fimAv_*MT*78_*	27 (14.4)	0	0	0	**27 (24.8)**		<0.00001
*papAH*	50 (26.6)	0	**30 (57.7)**	**31 (39.2)**	19 (17.4)	<0.00001	0.0008
*papC*	51 (27.1)	0	**30 (57.7)**	**31 (39.2)**	20 (18.3)	<0.00001	0.0013
*papEF*	52 (27.7)	0	**31 (59.6)**	**32 (40.5)**	20 (18.3)	<0.00001	0.0007
*sfa/focDE*	10 (5.3)	0	0	0	**10 (9.2)**		0.0036
*afa/draBC*	20 (10.6)	0	10 (19.2)	**15 (19.0)**	5 (4.6)		0.0018
*yfcV*	104 (55.3)	16 (100)	52 (100)	**77 (97.5)**	27 (24.8)		<0.00001
**Toxin**							
*sat*	82 (43.6)	15 (93.8)	50 (96.2)	**74 (93.7)**	8 (7.3)		<0.00001
*cnf1*	25 (13.3)	0	**17 (32.7)**	**18 (22.8)**	7 (6.4)	0.0049	<0.00001
*hlyA*	25 (13.3)	0	**17 (32.7)**	**18 (22.8)**	7 (6.4)	0.0049	<0.00001
*hlyF*	31 (16.5)	0	0	0	**31 (28.4)**		<0.00001
*cdtB*	5 (2.7)	0	1 (1.9)	1 (1.3)	4 (3.7)		
*tsh*	12 (6.4)	0	0	0	**12 (11)**		0.0011
*vat*	17 (9)	0	0	0	**17 (15.6)**		0.00005
**Iron uptake**							
*iucD*	132 (70.2	15 (93.8)	50 (96.2)	**74 (93.7)**	58 (53.2)		<0.00001
*iutA*	134 (71.3)	15 (93.8)	50 (96.2)	**74 (93.7)**	60 (55)		<0.00001
*iroN*	32 (17)	0	0	0	**32 (29.4)**		<0.00001
*fyuA*	128 (68.1)	16 (100)	52 (100)	**79 (100)**	49 (45)		<0.00001
*chuA*	121 (64.4)	16 (100)	52 (100)	**79 (100)**	42 (38.5)		<0.00001
**Capsule**							
*kpsM II*	106 (56.4)	15 (93.8)	49 (94.2)	**73 (92.4)**	33 (30.3)		<0.00001
*kpsM II-K2*	13 (6.9)	0	7 (13.5)	**11 (13.9)**	2 (1.8)		0.0015
*kpsM II-K5*	83 (44.1)	15 (93.8)	42 (80.8)	**62 (78.5)**	21 (19.3)		<0.00001
*neuC-K1*	10 (5.3)	0	0	0	**10 (9.2)**		0.0036
*kpsM III*	2 (1.1)	0	0	0	2 (1.8)		
**Miscellaneous**							
*cvaC*	21 (11.2)	0	0	0	**21 (19.3)**		<0.00001
*iss*	23 (12.2)	0	0	0	**23 (21.1)**		<0.00001
*traT*	139 (73.9)	13 (81.3)	42 (80.8)	**64 (81)**	75 (68.8)		0.0422
*ibeA*	12 (6.4)	0	0	0	**12 (11)**		0.0011
*malX*	113 (60.1)	16 (100)	52 (100)	**79 (100)**	34 (31.2)		<0.00001
*usp*	101 (53.7)	16 (100)	52 (100)	**79 (100)**	22 (20.2)		<0.00001
*ompT*	126 (67)	16 (100)	52 (100)	**79 (100)**	47 (43.1)		<0.00001
ExPEC status	108 (57.4)	15 (93.8)	50 (96.2)	**73 (92.4)**	35 (32.1)		<0.00001
UPEC status	99 (52.7)	16 (100)	52 (100)	**77 (97.5)**	22 (20.2)		<0.00001
APEC status	24 (12.8)	0	0	**0**	**24 (22)**		<0.00001

a *P-values (by Fisher's exact test) are shown where P < 0.05*.

*Significant differences are indicated in bold*.

The ST131 isolates showed a higher VF-encoding gene score (mean 13.367) compared with the non-ST131 isolates (mean 7.661) (*P* < 0.001). However, four isolates belonging to clones B2-CH38-15-ST95 and B2-CH38-294-ST95 were those with the highest number of virulence genes (mean 21.000).

Nineteen VF-encoding genes (*papAH, papC, papEF, afa/draBC, yfcV, sat, cnf1, hlyA, iucD, iutA, fyuA, chuA, kpsM II, kpsM II-K2, kpsM II-K5, traT, malX, usp*, and *ompT*) were significantly associated with ST131 isolates, whereas that 10 (*fimAv*_MT78_, *sfa/focDE, hlyF, tsh, vat, iroN, kpsM II-K1, cvaC, iss, and ibeA*) were significantly associated with non-ST131 isolates.

The C2 subclade isolates showed a higher virulence score (mean 14.250) compared with C1-M27 isolates (mean 10.875) (*P* < 0.001). The genes *papAH, papC, papEF, cnf1, and hlyA* were associated with the C2 subclade isolates.

The most prevalent virotypes identified in ST131 isolates were A (8 isolates), C2 (25), E (18) and F (14) and a new virotype similar to A (virotype A-like) displayed by seven isolates. Further, a second new virotype similar to E (virotype E-like) was found in one isolate (Table S1). The virotype A was found more frequently among Lucus Augusti isolates (*P* = 0.0280), while virotype F was more frequent among Beaujon isolates (*P* = 0.0293).

## Discussion

The management of urinary tract and bloodstream infections due to *E. coli* has been complicated by the emergence of multidrug-resistance, especially of that related to the expansion of high-risk clones such as ST131 (Nicolas-Chanoine et al., 2008; de Toro et al., 2017). Since 2006, the prevalence of ESBL-EC among *E. coli* causing bacteremia has raised in Lucus Augusti hospital. This increase has been due to the spread of the multidrug-resistant ST131 subclade C2 associated with the production of CTX-M-15. Thus, the number of ESBL-EC isolates increased from 1.0% during 2000–2005 to 5.5% during 2006–2011. While during the first period 0% of the ESBL-EC isolates belonged to subclade C2, during the second period this subclone represented 39.8% (Mamani et al., 2019). A similar situation has been reported in France in different hospitals (Brisse et al., 2012; Sauget et al., 2016) and worldwide (Peirano et al., 2012).

The main change with respect to previous studies conducted at the Lucus Augusti hospital, is the emergence of isolates producing CTX-M-27. Indeed, this enzyme was not produced by any of the 105 ESBL-EC isolates recovered from extraintestinal infections between 2006 and 2007 (Blanco et al., 2009) and by any of the 92 ESBL-EC bloodstream isolates collected from 2001 to 2011 (Mamani et al., 2019) and only by one of 47 ST131 ESBL-EC isolated from urinary tract infections in 2012 (Dahbi et al., 2013). Furthermore, the CTX-M-27 was only detected in one of the 44 Spanish hospitals analyzed in a study carried out in 2006 Díaz et al. (2010), in one of 94 clinical ESBL-EC collected during 2008 in Vall d'Hebron hospital of Barcelona (Coelho et al., 2011), and in none of the 92 ESBL-EC obtained in eight Spanish hospitals during 2010 and 2011 (Merino et al., 2016). In the present study, CTX-M-27 was significantly more frequent in Beaujon (15.6%) than in Lucus Augusti (6.5%). However, the Beaujon's CTX-M-27 percentage appeared as remarkably higher than those found before 2014 (4.5–5.4%) in other 19 hospitals located as Beaujon in the Paris area (Robin et al., 2017; Surgers et al., 2019). Inversely, this percentage was closer to that found between 2014 and 2017 in 24 pediatric centers located in six French regions, i.e., 12.4% among 251 ESBL-EC isolated from febrile urinary tract infections (FUTIs) (Birgy et al., 2020). In addition, it has to be noted that CTX-M-27-producing isolates had already been found in 2012 in the feces of children in day care centers (DDCs) in France (Blanc et al., 2014) and also in feces of patients hospitalized in Madrid during a European survey conducted between 2014 and 2015 (Merino et al., 2018).

The increased prevalence of the CTX-M-27 in the two hospitals enrolled in the present study was mainly due to the expansion of cluster C1-M27 since 17 of the 21 positive CTX-M-27 isolates belonged to this cluster. The four remaining isolates belonged to three different STs, including ST38 (2 isolates), ST90 and ST1193. In the FUTI study, Birgy et al. (2020) also showed that their 31 CTX-M-27-producing isolates mostly belonged to cluster C1-M27 (10 isolates) and ST38 (8 isolates), and also that two belonged to ST1193.

The two ST1193 isolates identified in the present study belonged to clonotype CH14-64 and serotype O75:HNM and were positive for CTX-M-14 (the isolate from Spain) and CTX-M-27 (the isolate from France). As far as we know, this is the first report of the new emerging global clone ST1193 among clinical ESBL-EC isolates from Spain. Inversely, the ST1193 has already been described in France and shown producing either CTX-M-15 or CTX-M-27 in the pediatric FUTIs study (Birgy et al., 2020) and CTX-M-14 among the fecal isolates obtained from children in DCCs in France (Blanc et al., 2014). ST1193 has also been detected in two of 243 third-generation-cephalosporin-resistant *E. coli* isolates obtained from patients with bloodstream infection in Denmark during 2015 (Roer et al., 2018), in three of 51 clinical ESBL-EC isolated in Germany during 2015 and 2016 (Valenza et al., 2019) and in 11 of 225 cefotaxime-resistant *E. coli* isolated from UTIs in South-West England during 2017 and 2018 (Findlay et al., 2019). ST 1193 is currently much more expanded in China (Xia et al., 2014; Wu et al., 2017) and the USA (Johnson et al., 2019; Tchesnokova et al., 2019).

Despite the emergence of ST1193 and ST131 cluster C1-M27, it is clear that ST131 subclade C2 associated to CTX-M-15 remains the most prevalent sublineage among ESBL-EC in the two hospitals studied here, as it is the case in most of the European hospitals (Merino et al., 2016; Sauget et al., 2016; Roer et al., 2018; Findlay et al., 2019; Valenza et al., 2019; Birgy et al., 2020). The C2 subclade isolates showed a higher virulence score (mean 14.250) compared with the subclade C1-M27 ST131 isolates (mean 10.875) (*P* < 0.001) and non-ST131 isolates (mean 7.661) (*P* < 0.001). Interestingly, the *papAH, papC, papEF, cnf1*, and *hlyA* genes were associated with the C2 subclade isolates, which mostly displayed the vitotypes A, A-like, C2, E and F. The virotype A-like is new and differs from the virotype A in the type of capsular *kpsM II* gene that is *K*5 instead of *K*2 (Dahbi et al., 2014). In future studies, it would be very interesting to determine the whole genome sequence of the C2 subclade isolates belonging to the new virotype A-like.

Of note, four isolates belonging to clones B2-CH38-15-ST95 and B2-CH38-294-ST95 were those with the highest number of VF-encoding genes (mean 21.000). These four UTI isolates were classified as APEC and could be of avian origin and foodborne pathogens (Vincent et al., 2010; Singer, 2015; Liu et al., 2018). ST95 is one of the most frequently ST identified among *E. coli* causing human extraintestinal infections, but it is rarely producer of ESBL enzymes (Kallonen et al., 2017). Nevertheless, recently Birgy et al. (2020) detected nine ST95 isolates among 251 ESBL-EC from pediatric FUTIs. The combination of so many virulence genes and resistance-encoding genes in this successful ST is very worrying.

## Conclusions

Despite the enormous genetic diversity observed in our ESBL-EC collection (71 clones amongst 188 ESBL-EC), it can be concluded that the majority of the isolates belong to only three clonal complexes (CC10, CC23, and CC131) and that ST131 subclade C2 associated with the production of CTX-M-15 remains the most prevalent *E. coli* lineage among the ESBL-EC isolates identified in the studied Spanish and French hospitals.

## Data Availability Statement

All datasets generated for this study are included in the article/Supplementary Material.

## Author Contributions

S-CF-S, VG, MD, NM, MA, IG-M, JEB, and MB undertook the laboratory work. M-HN-C and JB conceived the concept for the paper and designed the experiments. All authors provided critical input, contributed to the writing of the manuscript, and have approved the final version.

### Conflict of Interest

The authors declare that the research was conducted in the absence of any commercial or financial relationships that could be construed as a potential conflict of interest.

## Supplementary Material

Table S1 Characteristics of 188 extended-spectrum β-lactamase-producing *Escherichia coli* (ESBL-EC) collected in 2015 in Lucus Augusti University hospital (Lugo, Spain) and AP-HP Beaujon hospital (Clichy, France).
